# Clear cell variant of diffuse large B-cell lymphoma: a case report

**DOI:** 10.1186/1752-1947-5-182

**Published:** 2011-05-13

**Authors:** Suzana Manxhuka-Kerliu, Gordana Petrusevska, Irma Kerliu, Emrush Kryeziu, Fehmi Ahmeti, Emine Devolli-Disha, Vjollca Sahatciu-Meka, Sadushe Loxha, Labinot Shahini

**Affiliations:** 1 Faculty of Medicine, Institute of Pathology, University of Prishtina, Mother Theresa Street NN, 10 000, Prishtina, Kosovo; 2 Faculty of Medicine, Institute of Pathology, Ss Cyril and Methodius University of Skopje, Vodnjanska NN, 1000, Skopje, Former Yugoslav Republic of Macedonia; 3 Massachusetts College of Pharmacy and Health Sciences (MCPHS), 179 Longwood Avenue, Boston, MA 02115, USA; 4 Hematology Clinic, University Clinical Center of Kosovo, Mother Theresa Street NN, 10 000, Prishtina, Kosovo; 5 Radiology Clinic, Faculty of Medicine, Institute of Pathology, University Clinical Center of Kosovo, Mother Theresa Street NN, 10 000, Prishtina, Kosovo; 6 Faculty of Medicine, University Clinical Center of Kosovo, Mother Theresa Street NN, 10 000, Prishtina, Kosovo

## Abstract

**Introduction:**

Diffuse large B-cell lymphoma is a diffuse proliferation of large neoplastic B lymphoid cells with a nuclear size equal to or exceeding the normal macrophage nuclei. We report a case of a clear cell variant of diffuse large B-cell lymphoma involving a lymph node in the neck, which was clinically suspected of being metastatic carcinoma.

**Case presentation:**

A 39-year-old Caucasian ethnic Albanian man from Kosovo presented with a rapidly enlarging lymph node in his neck, but he also disclosed B symptoms and fatigue. A cytological aspirate of the lymph node revealed pleomorphic features. Our patient underwent a cervical lymph node biopsy (large excision). The mass was homogeneously fish-flesh, pale white tissue replacing almost the whole structure of the lymph node. The lymph node biopsy showed a partial alveolar growth pattern, which raised clinical suspicion that it was an epithelial neoplasm. With regard to morphological and phenotypic features, we discovered large nodules in diffuse areas, comprising large cells with slightly irregular nuclei and clear cytoplasm admixed with a few mononuclear cells. In these areas, there was high mitotic activity, and in some areas there were macrophages with tangible bodies. Staining for cytokeratins was negative. These areas had the following phenotypes: cluster designation marker 20 (CD20) positive, B-cell lymphoma (Bcl)-2-positive, Bcl-6^-^, CD5^-^, CD3^-^, CD21^+ ^(in alveolar patterns), prostate-specific antigen-negative, human melanoma black marker 45-negative, melanoma marker-negative, cytokeratin-7-negative and multiple myeloma marker 1-positive in about 30% of cells, and exhibited a high proliferation index marker (Ki-67, 80%).

**Conclusion:**

According to the immunohistochemical findings, we concluded that this patient has a clear cell variant of diffuse large B-cell lymphoma of activated cell type, post-germinal center cell origin. Our patient is undergoing R-CHOP chemotherapy treatment.

## Introduction

Diffuse large B-cell lymphoma (DLBCL) displays striking heterogeneity at the clinical, genetic and molecular levels [1]. DLBCL is the most common type of lymphoid tumor worldwide. This category was included in both the Revised European American Lymphoma (REAL) [2] classification system and the World Health Organization (WHO) classifications of 2001 [3] and 2008 [4], with the aim of lumping together all malignant lymphomas characterized by the large size of the neoplastic cells of B-cell derivation as well as by an aggressive clinical presentation and the need for highly effective chemotherapy regimens [5]. These tumors are detected as primary or secondary forms at both the nodal and extra-nodal levels in immune-competent hosts as well as in patients with different types of immune-suppression. They display significant variability in terms of cell morphology and clinical findings, which justifies the identification of variants and subtypes [5]. DLBCL is a diffuse proliferation of large neoplastic B lymphoid cells with a nuclear size equal to or exceeding that of normal macrophage nuclei. However, even on the basis of simple histological examination, considerable heterogeneity can be seen, and several morphological variants have been described [3].

Immunophenotype, tissue microarray and molecular studies underline the extreme heterogeneity of DLBCLs and suggest a sub-classification of the tumor on the basis of the identification of different pathogenic pathways; this might have much greater relevance than pure morphology for precise prognostic previsions and the adoption of *ad hoc *therapies. Recent reports regarding the pathobiology of DLBCLs are reviewed in light of these authors' experience, with the aim of contributing to the existing debate on the topic [6,7].

DLBCL is the most common type of non-Hodgkin's lymphoma. The International Prognostic Index is useful in predicting the outcomes of patients with DLBCL. The discovery of specific genetic alterations and the assessment of protein expression led to the identification of multiple, novel, single molecular markers capable of predicting the outcomes of patients with DLBCL independently of clinical variables [8]. However, much confusion exists in the literature regarding the importance of different prognostic biomarkers and their applicability in routine practice [9-14].

## Case presentation

A 39-year-old Caucasian ethnic Albanian man from Kosovo presented with rapidly enlarging lymph nodes in his neck, but he also disclosed B symptoms and fatigue. A peripheral blood examination revealed no pathological changes. A cytological aspirate of his lymph node disclosed pleomorphic features. Our patient underwent a cervical lymph node biopsy (large excision).

According to the first biopsy findings, based on hematoxylin and eosin-stained slides created by co-authors (SL and LSH), the diagnosis was suspected of being a cervical lymph node metastatic carcinoma; therefore, it was investigated for primary carcinoma, which was not identified. Clinically and radiographically, the mediastinum was clear. This case has been reviewed by the authors (SMK and GP), who arrived at the final diagnosis of a clear cell variant of DLBCL.

### Macroscopic findings

The mass was homogeneously fish-flesh, pale white tissue replacing almost the whole structure of the lymph node. There was no other tumor mass in the body or in the mediastinum.

### Histological and phenotypic findings

**Figure 1:**
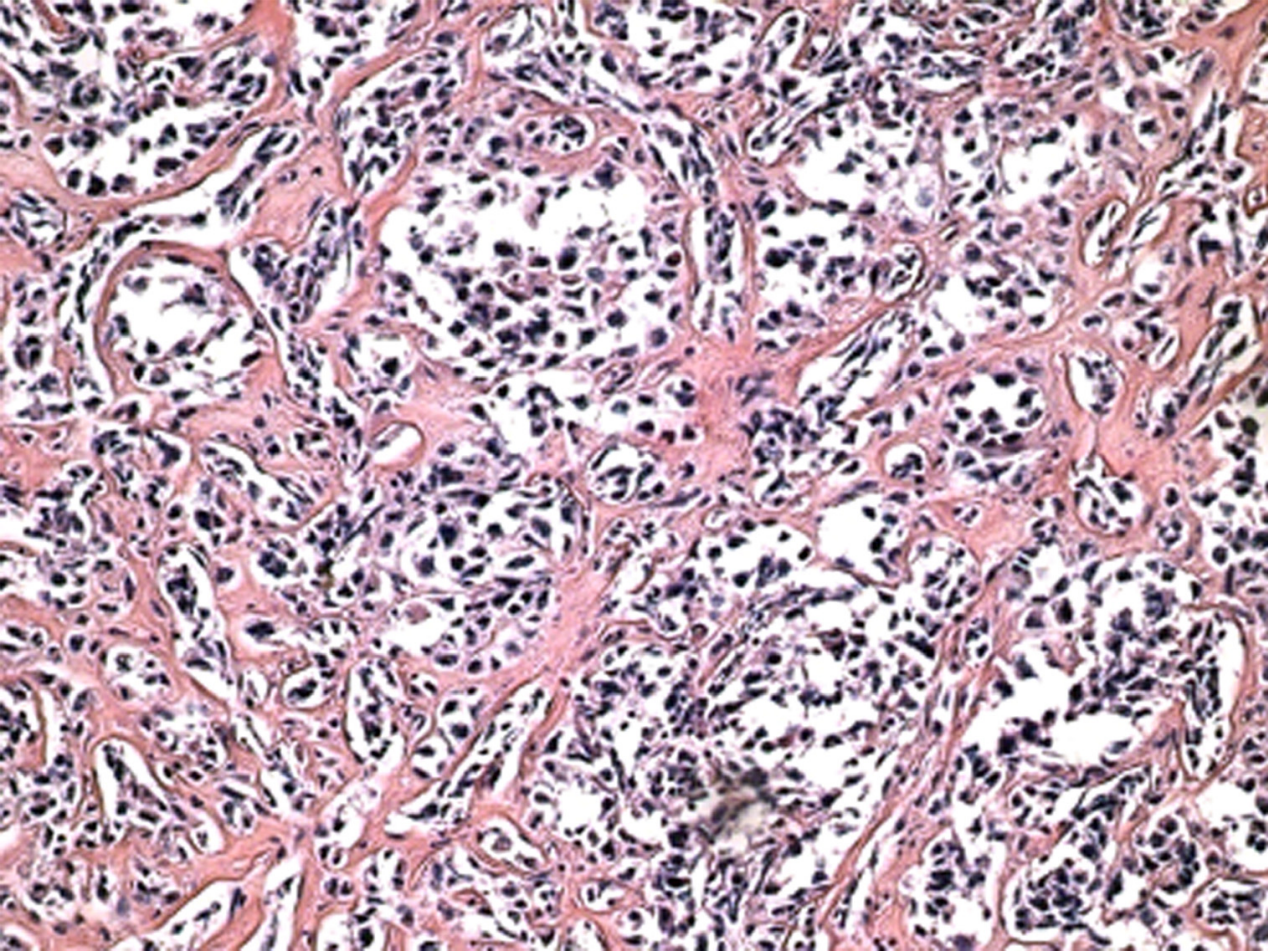
**Marked sclerosis and hyalinization in diffuse large B-cell lymphoma (hematoxylin and eosin stain; original magnification, × 20)**.

**Figure 2:**
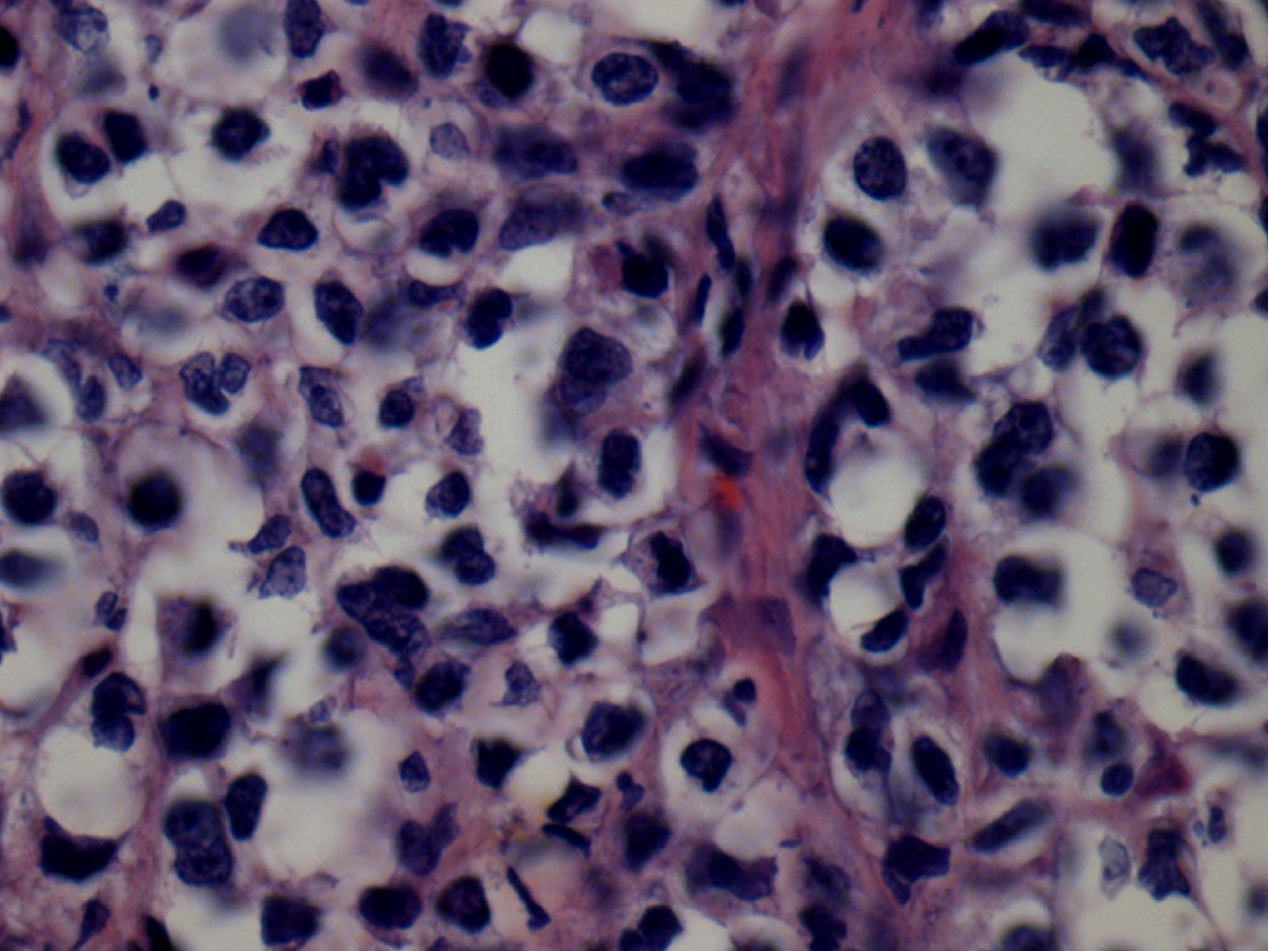
**Sheets of large cells with abundant pale cytoplasm separated by collagenous fibrosis**. Nuclei are round (centroblast-like) or sometimes multi-lobulated (hematoxylin and eosin stain; original magnification, × 40).

**Figure 3:**
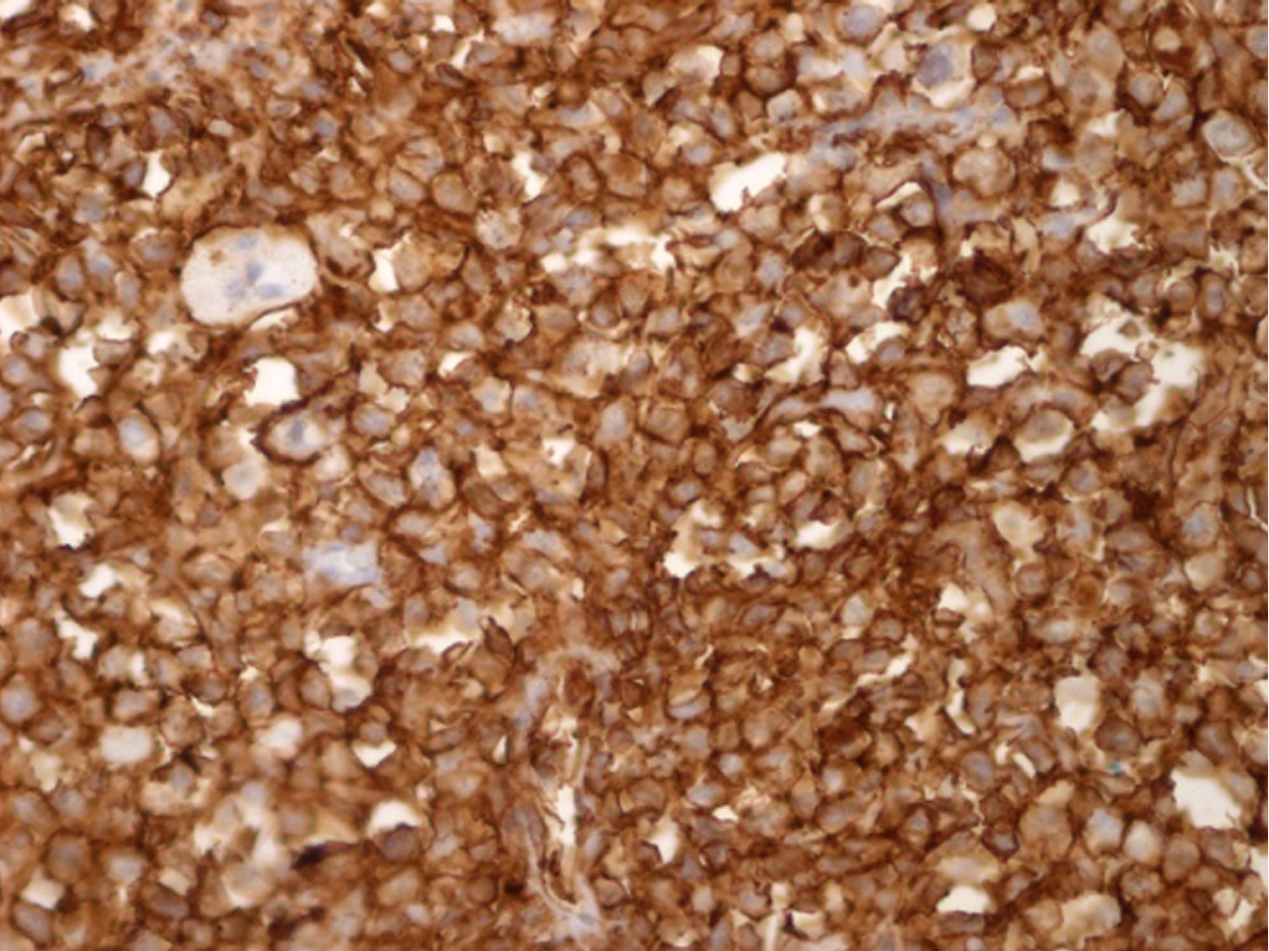
**CD20**^**+ **^**expressed strong positivity (CD 20 stain; original magnification, × 40)**.

**Figure 4:**
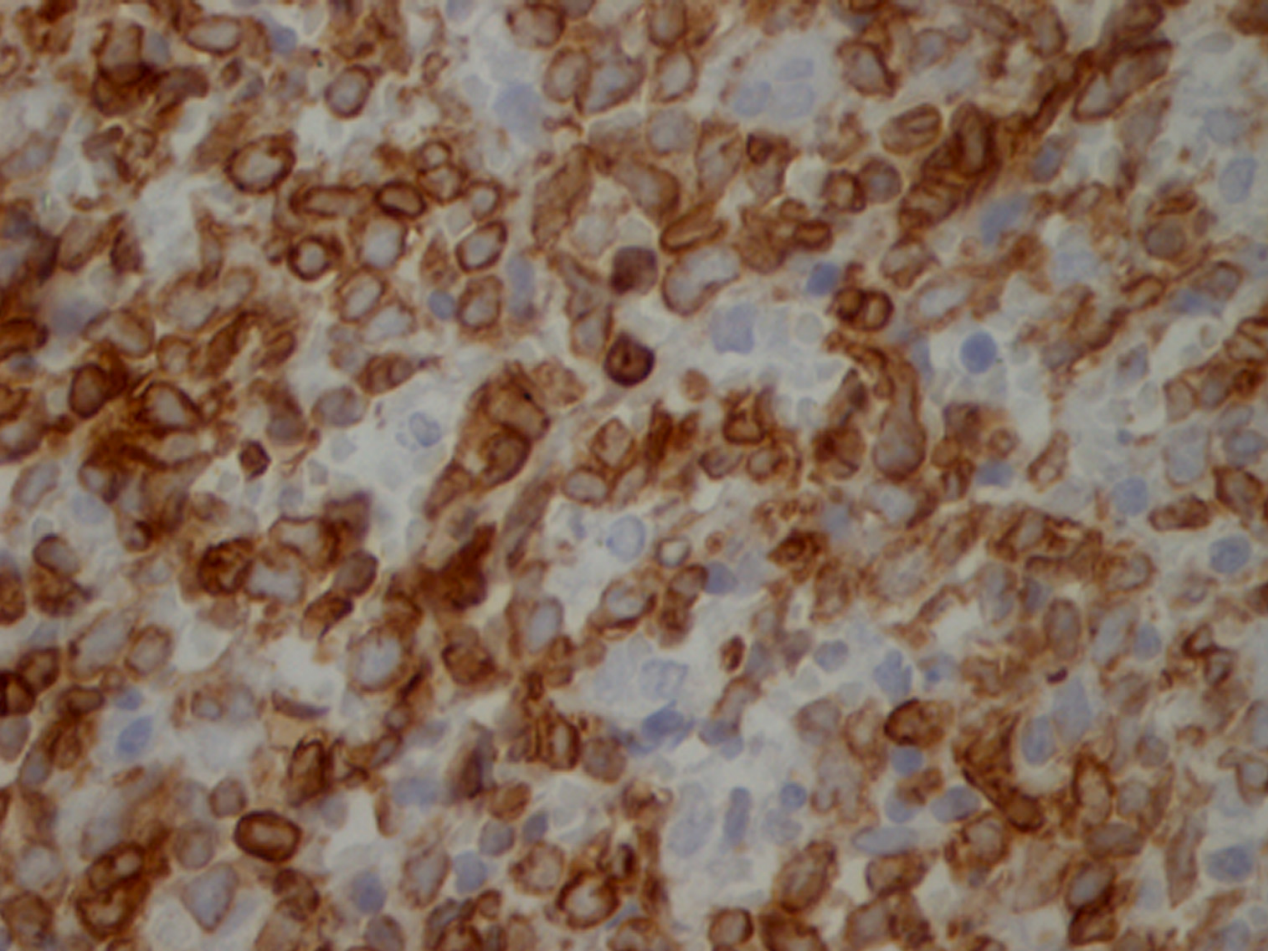
**Bcl-2**^**+ **^**expressed cytoplasmic staining (Bcl-2 stain; original magnification, × 40)**

**Figure 5:**
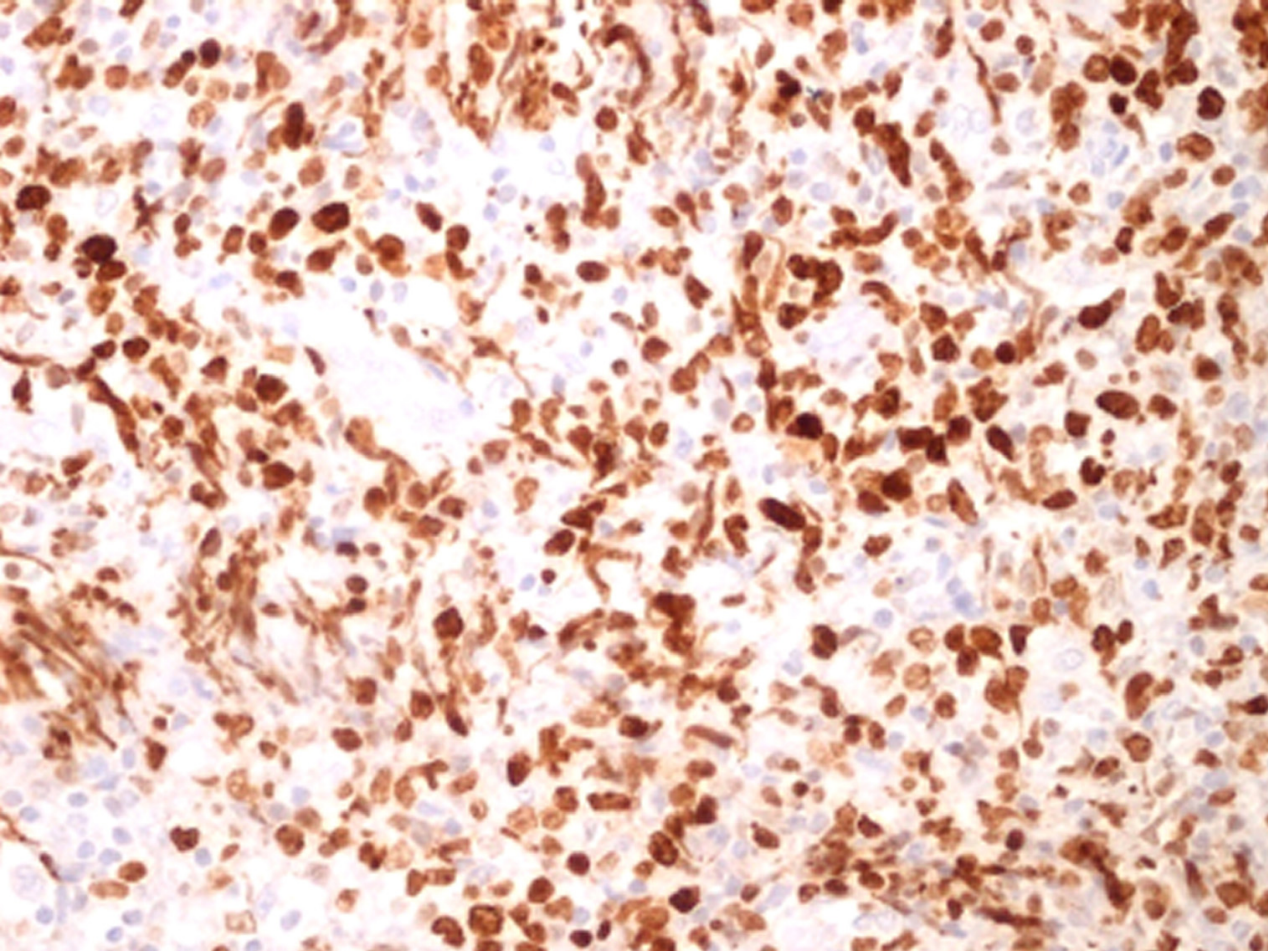
**Ki-67**^**+ **^**expressed a high proliferation index (Ki-67 stain, 80%, × 20)**.

The lymph node biopsy showed a partially alveolar growth pattern, marked sclerosis and hyalinization (Figure 1), which raised clinical suspicions of an epithelial neoplasm. The morphological and phenotypic features comprised large nodules in diffuse areas, composed of large cells with slightly irregular nuclei and clear cytoplasm admixed with a few mononuclear cells, as well as sheets of large cells with abundant pale cytoplasm separated by collagenous fibrosis. The nuclei were round (centroblast-like) or sometimes multi-lobulated (Figure 2). These areas displayed high mitotic activity, and some areas contained macrophages with tangible bodies. Staining for cytokeratins (CK) was negative. These areas disclosed the following phenotype: cluster designation marker 20 (CD20) expressed strong positivity (Figure 3), B-cell lymphoma (Bcl)-2 expressed cytoplasmic staining (Figure 4), Bcl-6^-^, CD5^-^, CD3^-^, CD21^+ ^(in alveolar patterns), prostate-specific antigen-negative (PSA^-^), human melanoma black marker 45-negative (HMB45^-^), melanoma marker-negative (Melan^-^), CK7^- ^and multiple myeloma marker 1-positive (MUM1^+^) in about 30% of cells and Ki-67 expressed a high proliferation index of 80%. (Figure 5).

On the basis of the histological examination, the differential diagnosis of the tumor needed to include other lympho-proliferative conditions in which large B-cells could be observed. Our first thought was the possibility of primary mediastinal large B-cell lymphoma (PMBCL), but clinically no tumor mass was found in the mediastinum. Radiography of the mediastinum did not show any pathological change.

We also considered possible metastases from prostate carcinoma or malignant melanoma, as well as mesenchymal large-cell neoplasms or undifferentiated large-cell carcinoma. Therefore, we performed immunohistochemistry (IHC) to define the histological type of the tumor. The technique used in our case was the avidin-biotin complex (ABC) as a standard IHC method.

The IHC results excluded clinical suspicions of a metastatic tumor. The differential diagnosis following the first round of IHC included uncommon, undifferentiated large-cell carcinoma, malignant melanoma, undifferentiated mesenchymal large-cell neoplasms and prostate carcinoma.

In the second round of IHC, we considered the possibility of DLBCL. (The morphological variants are centroblastic, immunoblastic, T-cell- and histiocyte-rich, anaplastic, plasmablastic, DLBCL-anaplastic lymphoma kinase-positive and PMBCL.) Our final diagnosis was of a clear cell variant of DLBCL.

## Discussion

All large B-cell lymphomas have been lumped together into two categories in the REAL classification published in 1994 [2]: DLBCL and PMBCL. In the WHO classification of 2001 [3], and even in the new WHO classification of 2008 [4], the most convincing variants of DLBCL were therefore separated based on the belief that these variants represent distinct clinico-pathologic entities [15].

Our case had to be differentiated from other variants of DLBCL, such as T-cell histiocyte-rich large B-cell lymphoma (TCHRLBCL), which shows CD20^+^, CD30^-^, CD15^-^, almost no small CD20^+ ^or immunoglobulin D-positive (IgD^+^) B-cells and often more CD8^+ ^than CD4^+ ^T-cells in the background. We also had to differentiate our case from PMBCL.

Employing various immunohistochemical antibodies, such as CD10, CD138, anti-Bcl-2, anti-Bcl-6, MUM1 and anti-p53, several groups have tried to sub-classify DLBCL into the germinal center B-cell-like DLBCL (GCB-DLBCL) and activated B-cell-like DLBCL (ABC-DLBCL) sub-groups, with comparable differences in clinical behavior [16]. Alizadeh *et al*. [17] identified two molecularly distinct forms of DLBCL which had gene expression patterns indicative of different stages of B-cell differentiation. One type expressed genes characteristic of GCB-DLCBL, and the second type expressed genes normally induced during *in vitro *activation of peripheral blood B-cells (ABC-DLBCL). Patients with GCB-DLCBL had significantly better overall survival than those with ABC-DLBCL [18,19].

The patients with GCB-DLCBL had better prognosis than the non-GCB subtype. Both ABC-DLBCL and GCB-DLBCL show a significant improvement of overall survival after rituximab, cyclophosphamide, doxorubicin, Oncovin (vincristine sulfate) and prednisolone (R-CHOP) chemotherapy treatment [20]. Our patient with ABC-DLBCL underwent R-CHOP treatment and is alive.

Over-expression of Bcl-6 protein caused by *Bcl-6 *gene rearrangement may play some important roles in the development and/or progression of a subset of DLBCL [21]. The group with pattern B (ABC-DLBCL) demonstrated more frequent expression of Ki-67, cyclin D3 and geminin, and showed higher proliferation activity than the group with pattern A (GCB-DLBCL). These findings suggest that high proliferation activity of tumors with pattern B may be associated with aggressive tumor behavior and poor clinical outcomes in patients with DLBCL [22].

Commonly observed genetic abnormalities that likely contribute to pathogenesis include translocation of *BCL-6, BCL-2, c-Myc *and *FAS *(CD95) mutations and aberrant somatic hyper-mutation. Additional novel therapies under investigation include those targeting BCL6 and BCL2. Also, the development of novel monoclonal antibody-based therapies is underway.

PMBCL has been thought of as a special subtype of DLBCL. Its distinct clinical presentation in younger patients, with a female predominance, has led to the suspicion that it constitutes a unique entity. However, a reliable distinction from DLBCL has remained elusive [23].

The subdivision of grade 3 follicular lymphoma (FL3) into the cytologic subtypes of 3a, 3b, and follicular large cleaved cell lymphoma (FLC) does not appear to be clinically important. However, to prevent its misclassification as a low-grade follicular lymphoma, FLC should be recognized and considered as a morphologic subtype of FL3 for clinical purposes. Finally, patients with FL3 with a significant diffuse component (>50%) have an inferior survival that is similar to the survival of those with DLBCL [24].

In children, the Burkitt lymphoma (BL) and DLBCL subtypes probably do not differ clinically, although the differential diagnosis between BL and DLBCL may theoretically appear clear-cut. In adults, daily practice shows the existence of cases that have immuno-phenotypic and cytogenetic morphological features that are intermediate between DLBCL and BL, and thus cannot be classified into either of these categories with certainty [25].

The overlap between BL and DLBCL has been discussed, including mediastinal "gray zone" lymphoma and other lymphomas with atypical immuno-phenotypes. These overlapping lymphoma types include the gray zone around nodular lymphocyte-predominant Hodgkin's lymphoma (NLPHL), TCHRLBCL, classical Hodgkin's lymphoma (cHL), Epstein-Barr virus-positive lymphomas, lymphomas occurring in patients with human immunodeficiency virus, post-transplant lympho-proliferative disorder-related B-cell lympho-proliferations and DLBCLs with an unusual immuno-phenotype. It has become clear that the "double-hit" cases (the combination of a c-Myc breakpoint with mostly BCL2 breakpoints and other recurrent chromosomal breakpoints), often with distinct morphological features of BL, should fall into a novel category of "B-cell lymphoma, unclassifiable, with features intermediate between DLBCL and BL."

The main issue addressed during the workshop of the XIV meeting of the European Association for Hematopathology was to define criteria to reliably distinguish entities such as NLPHL, TCHRLBCL and the gray zones between cHL and DLBCL, mainly, TCHRLBCL in the lymph nodes [26].

## Conclusion

"Gray zone lymphoma", a term which has been used to denote a group of various types of lymphomas with overlapping histological, biological and clinical features, remains a diagnostic problem for pathologists. On the basis of our IHC findings, we have concluded that the diagnosis in the present case is a clear cell variant of DLBCL of activated cell type, post-germinal center cell origin. Our patient is alive and undergoing R-CHOP chemotherapy treatment. Increased molecular understanding of the heterogeneous subsets within DLBCL will likely improve the current treatment of patients with DLBCL by identifying rational therapeutic targets in specific disease subtypes.

## Abbreviations

ABC: avidin-biotin complex; ABC-DLBCL: activated B-cell-like DLBCL; Bcl: B-cell lymphoma; BL: Burkitt lymphoma; CD: cluster designation marker; cHL: classical Hodgkin's lymphoma; CK: cytokeratin; DLBCL: diffuse large B-cell lymphoma; FL: follicular lymphoma; FL3: grade 3 follicular lymphoma; GCB-DLBCL: germinal center B-like DLBCL: IHC: immunohistochemistry; Ki-67: proliferation index marker; MUM: multiple myeloma marker; NLPHL: nodular lymphocyte-predominant Hodgkin's lymphoma; PMBCL: primary mediastinal B-cell lymphoma; R-CHOP: rituximab, cyclophosphamide, doxorubicin, Oncovin and prednisolone; REAL: Revised European American Lymphoma classification; TCHRLBCL: T-cell histiocyte-rich large B-cell lymphoma; WHO: World Health Organization;

## Consent

Written informed consent was obtained from the patient for publication of this case report and any accompanying images. A copy of the written consent is available for review by the Editor-in-Chief of this journal.

## Competing interests

The authors declare that they have no competing interests.

## Authors' contributions

All of the authors were involved in the conception of the case report, the data collection and the literature review as well as in writing the manuscript. SMK performed the histological examination of the lymph node and was a major contributor in writing the manuscript. GP performed the immunohistochemical examination and interpretation. IK and LSH reviewed the literature. EK, FA, EDD and VSM analyzed and interpreted the clinical data. SL performed data collection. All authors read and approved the final manuscript.
